# Electrophysiological Properties of Induced Pluripotent Stem Cell-Derived Midbrain Dopaminergic Neurons Correlate With Expression of Tyrosine Hydroxylase

**DOI:** 10.3389/fncel.2022.817198

**Published:** 2022-03-23

**Authors:** Aleksandar Rakovic, Dorothea Voß, Franca Vulinovic, Britta Meier, Ann-Katrin Hellberg, Carla Nau, Christine Klein, Enrico Leipold

**Affiliations:** ^1^Institute of Neurogenetics, University of Lübeck, Lübeck, Germany; ^2^Department of Anesthesiology and Intensive Care, Center of Brain, Behavior and Metabolism (CBBM), University of Lübeck, Lübeck, Germany

**Keywords:** dopaminergic neurons, tyrosine hydroxylase, differentiation, Parkinson’s disease, patch-clamp, electrophysiology, iPSC

## Abstract

Induced pluripotent stem cell (iPSC)-based generation of tyrosine hydroxylase-positive (TH^+^) dopaminergic neurons (DNs) is a powerful method for creating patient-specific *in vitro* models to elucidate mechanisms underlying Parkinson’s disease (PD) at the cellular and molecular level and to perform drug screening. However, currently available differentiation paradigms result in highly heterogeneous cell populations, often yielding a disappointing fraction (<50%) of the PD-relevant TH^+^ DNs. To facilitate the targeted analysis of this cell population and to characterize their electrophysiological properties, we employed CRISPR/Cas9 technology and generated an mCherry-based human TH reporter iPSC line. Subsequently, reporter iPSCs were subjected to dopaminergic differentiation using either a “floor plate protocol” generating DNs directly from iPSCs or an alternative method involving iPSC-derived neuronal precursors (NPC-derived DNs). To identify the strategy with the highest conversion efficiency to mature neurons, both cultures were examined for a period of 8 weeks after triggering neuronal differentiation by means of immunochemistry and single-cell electrophysiology. We confirmed that mCherry expression correlated with the expression of endogenous TH and that genetic editing did neither affect the differentiation process nor the endogenous TH expression in iPSC- and NPC-derived DNs. Although both cultures yielded identical proportions of TH^+^ cells (≈30%), whole-cell patch-clamp experiments revealed that iPSC-derived DNs gave rise to larger currents mediated by voltage-gated sodium and potassium channels, showed a higher degree of synaptic activity, and fired trains of mature spontaneous action potentials more frequently compared to NPC-derived DNs already after 2 weeks in differentiation. Moreover, spontaneous action potential firing was more frequently detected in TH^+^ neurons compared to the TH^–^ cells, providing direct evidence that these two neuronal subpopulations exhibit different intrinsic electrophysiological properties. In summary, the data reveal substantial differences in the electrophysiological properties of iPSC-derived TH^+^ and TH^–^ neuronal cell populations and that the “floor plate protocol” is particularly efficient in generating electrophysiologically mature TH^+^ DNs, which are the most vulnerable neuronal subtype in PD.

## Introduction

Parkinson’s disease (PD) is the second most common neurodegenerative disorder and characterized by a combination of motor and non-motor symptoms that are barely noticeable in early disease stages but gradually increase in intensity as the disease progresses (Bloem et al., 2021). At the cellular level, PD signs are linked to a progressive loss of dopaminergic neurons (DNs) in the *substantia nigra pars compacta* (SNpc), resulting in extensive alterations of the neurochemistry in the midbrain (Blaszczyk, 2016; Magrinelli et al., 2016; Bloem et al., 2021). The reasons for the selective vulnerability of SNpc DNs in PD conditions are not well understood.

The generation of induced pluripotent stem cells (iPSCs) from patient-derived blood cells or fibroblasts using cellular reprogramming techniques (Takahashi et al., 2007) enabled the generation of patient-specific *in vitro* disease models, including PD-relevant DN cultures, suitable for analyzing disease mechanisms at cellular and molecular levels (Shi et al., 2017) as well as drug screening (Garcia-Leon et al., 2019; Kouroupi et al., 2020). Among the various established differentiation procedures (Cooper et al., 2010; Kriks et al., 2011; Kirkeby et al., 2012; Sanchez-Danes et al., 2012), the “midbrain floor plate protocol” (Kriks et al., 2011), which is based on early patterning of differentiating cells toward a floor plate stage, is considered one of the most effective protocols for generating human DNs from iPSCs (Weykopf et al., 2019). An attractive alternative which bypasses the continuous and cumbersome generation of iPSCs and thus significantly reduces the time required to obtain DN cultures utilizes iPSC-derived neuronal precursor cells (NPCs) as starting material for subsequent differentiation steps (Reinhardt et al., 2013). In contrast to iPSCs, NPCs are easier to handle because they exhibit robust immortal expansion, and their cultivation involves less manual manipulation.

Despite these promising developments, only between 30 and 80% of the cells in iPSC-derived DN cultures express pan-neuronal markers such as βIII-tubulin, of which again only 8–85% also express TH, the rate-limiting enzyme of dopamine synthesis, corresponding to proportions of 3–30% TH^+^ cells relative to all cells (Sundberg et al., 2013; Ishikawa et al., 2016; Awad et al., 2017; Beevers et al., 2017; Romero-Moya et al., 2017; Sheng et al., 2017). The most likely reasons for the variable yields of TH^+^ neurons are considered to be differences in the iPSC lines used and non-standardized, laboratory-specific handling of the cells (Mahajani et al., 2019). In addition, studies assessing the electrical excitability of human iPSC-derived DN cultures demonstrated that the cells are also very heterogenous with respect to their electrophysiological properties (Belinsky et al., 2011; Hartfield et al., 2014; Pre et al., 2014), most likely due to high proportions of immature neurons. This intrinsic inhomogeneity of iPSC-derived DNs remains a challenge as it adds variability to datasets in downstream applications and limits their interpretation (Calatayud et al., 2019; Laperle et al., 2020).

Recently, a series of elegant studies demonstrated how some of these limitations can be circumvented. These studies described the generation of TH reporter iPSC lines engineered to express fluorescent proteins such as mOrange (Calatayud et al., 2019), GFP (Hong and Daadi, 2019; Uberbacher et al., 2019) or RFP (Xia et al., 2017) which were introduced into the endogenous TH locus, right before TH’s stop codon using a CRISPR/Cas9-based editing strategy. Immunochemistry analysis and transcriptional assays confirmed the neuronal identity of TH-expressing (TH^+^) cells, however, the electrophysiological properties of TH^+^ and TH^–^ subpopulations were not evaluated in these studies. To fill this gap, we employed CRISPR/Cas9 technology to generate a mCherry-based human TH reporter iPSC line and subjected the cells to dopaminergic differentiation using either a direct route for differentiation of iPSC into DNs (Kriks et al., 2011) (iPSC-derived DNs) or an indirect route involving NPCs (NPC-derived DNs) as an intermediate step (Reinhardt et al., 2013). Using single-cell patch-clamp electrophysiology, we assessed for the first time the development of the electrophysiological properties of TH^+^ and TH^–^ neurons in both reporter cultures over a period of 8 weeks and demonstrate that mature electrophysiological properties of iPSC-derived DNs is correlated with endogenous TH expression and depends on the applied differentiation strategy.

## Materials and Methods

### Generation of a Tyrosine Hydroxylase Reporter Line

A human iPSC line (Zanon et al., 2017) generated from human foreskin fibroblasts (ATCC, CRL-2522) was used as starting material to engineer a TH-mCherry iPSC reporter line. To facilitate gene editing of CRL-2522 cells, we used plasmid pX330S-2 (a gift from Takashi Yamamoto; Addgene plasmid # 58778^1^; RRID:Addgene_58778) (Sakuma et al., 2014). Custom guide RNA (gRNA) targeting the sequence 5′- GTGCCATTGGCTAGGTGCA-3′ in exon 14 of the TH gene was cloned into the *Bbs*I sites as annealed oligonucleotides. The donor template for homology-directed repair (HDR) was generated using Gibson cloning as described previously (Calatayud et al., 2019). In brief, for the TH donor template, 1 kb-long homology arms (HA) were amplified from genomic DNA extracted from unmodified iPSCs and verified by Sanger sequencing. HA were inserted into the pBS-SK (−) vector using *Kpn*I and *Apa*I restriction sites for the 5′ HA and *Spe*I and *Xba*I restriction sites for the 3′ HA sites of pBS-SK (−). A sequence encoding the T2A peptide was fused to the 5′ end of the mCherry open reading frame. Finally, the pRex1-Neo-SV40 cassette was inserted between the *Xho*I and *Spe*I restriction sites of the plasmid.

Next, iPSCs were transfected with the HDR template and the pX330S-2 plasmid expressing the gRNA mentioned above. In total, one million iPSCs were disaggregated into single cells using Accutase (Stemcell) on the day of transfection. Disaggregated iPSCs were diluted in 100 μl of human stem cell nucleofector Kit 2 solution (Lonza Group Ltd., Switzerland) supplemented with 2 μg of pX330S-2 plasmid and 2 μg HDR template and transfected using the 2b Nucleofector device (Lonza Group Ltd., Switzerland). Transfected cells were plated onto Matrigel (BD)-coated 6-well culture plates containing mTeSR1 medium (Stemcell), supplemented with ROCK inhibitor Y-27632 (EMD Biosciences). On the following day, the medium was replaced with fresh mTeSR1 containing 50 μg/mL G418 (Sigma) and cells were maintained for 2 weeks. At that time, one half of each resistant colony was manually picked, resuspended in 1x PCR buffer containing Proteinase K and incubated for 50 min at 56°C followed by 10 min incubation at 95°C. Integration of the resistance cassette into the TH locus was verified by PCR. Positive colonies were expanded and cryopreserved.

To excise the selection cassette, positive iPSCs were transfected with 2 μg of a Cre recombinase-expressing plasmid (pCAG-Cre was a gift from Connie Cepko; Addgene plasmid # 13775^2^; RRID:Addgene_13775) (Matsuda and Cepko, 2007) diluted in 100 μl of human stem cell nucleofector Kit 2 solution using the 2b Nucleofector device (both Lonza Group Ltd., Switzerland). Subsequently, transfected cells were plated onto Matrigel (BD)-coated dishes in mTeSR1 (Stemcell), supplemented with ROCK inhibitor Y-27632 (EMD Biosciences Ltd.). One week post transfection, one half of each resistant colony was manually picked, resuspended in 1x PCR buffer containing Proteinase K and incubated 50 min at 56°C followed by 10 min incubation at 95°C. The colonies were analyzed for complete excision of the resistance cassette using a PCR-based strategy. Clones with successfully excised resistance cassette were expanded, the iPSC lines were comprehensively characterized including expression analyses of pluripotency markers, and cryopreserved.

To yield DN-cultures, unedited and edited iPSCs were subjected to dopaminergic differentiation following either a “floor plate differentiation method” or an alternative strategy involving iPSC-derived NPCs. The associated procedures are detailed in the Supplemental Material.

### Immunocytochemistry

Cells were fixed with 4% paraformaldehyde for 15 min at room temperature (RT) and permeabilized for 15 min in 0.1% NP-40 in phosphate-buffered saline (PBS). Cells were blocked using 1% of normal goat serum for 1 h at RT. The following antibodies were used: rabbit anti-OCT4 (Abcam), mouse anti-TRA-1-60 (Millipore), rabbit anti-NANOG (Stemgent), mouse anti-SSEA-4 (Millipore), rabbit anti-TH (Calbiochem), mouse anti-SOX2 (R&D Systems), mouse anti-mouse anti-Nestin (Santa Cruz), mouse anti-Pax6 (Santa Cruz). Corresponding Alexa Fluor-conjugated secondary antibodies were from Thermo Fisher. To label nuclei, coverslips were mounted using DAPI Fluoromount-G (SouthernBiotech). Images were acquired using a confocal microscope (Zeiss LSM710) operated by the ZEN software package. ImageJ (Schneider et al., 2012) and Adobe Photoshop CS6 (Adobe, United States) software were used for final image processing.

### Fluorescence-Activated Cell Sorting

Dopaminergic neuronal cultures differentiated from parental and TH-mCherry reporter iPSCs were harvested 50 days after starting the differentiation regime. Briefly, cells were washed with 1x PBS, detached using Accutase (Stemcell Technologies) and singularized by filtering through a 37 μm reversible cell strainer (Stemcell Technologies). Subsequently, cells were pelleted, resuspended in 1 ml of 1x PBS, and sorted on a BD FACSAria*™* III device (Becton, Dickinson and Company) to obtain mCherry-positive and mCherry-negative fractions.

### Western Blotting

Protein fractions from non-sorted, sorted mCherry-negative, and sorted mCherry-positive cells were isolated using RIPA buffer. Ten micrograms of protein from each fraction were separated on NuPAGE*™* 4–12%, Bis-Tris, 1.0 mm gels (Thermo Fisher Scientific) by SDS polyacrylamide gel electrophoresis and blotted. The membrane was probed using antibodies raised against TH (Merckmillipore), GAPDH (Cell Signaling), or β-actin (Sigma). For densitometric analyses, TotalLab software (Non-linear Dynamics) was used.

### High-Performance Liquid Chromatography

Dopaminergic neuronal cultures differentiated from TH-mCherry reporter iPSCs were harvested 50 days after starting the differentiation regime and sorted into mCherry-positive (TH^+^) and mCherry-negative (TH^–^) cells using FACS as mentioned above. Two million cells were harvested, pelleted, washed in 1x PBS, and lysed in 200 μL of dopamine extraction buffer (100 mM perchloric acid, 0.2 mM EDTA) using sonification (2 × 5 s, 50% amplitude, Bandelin). The total amounts of dopamine (DA), 3,4-dihydroxyphenylacetic acid (DOPAC), and homovanillic acid (HVA) were measured by means of high-performance liquid chromatography using a C18 column (Eurospher RP 18, particle size 5 pm, column size 250 mm × 4.0 mm) combined with subsequent electrochemical detection. In addition, three standard solutions each for DA, DOPAC, and HVA were prepared and subsequently measured to establish corresponding standard curves. The mobile phase was a degassed solution of 0.15 M sodium acetate buffer (pH 4.0) containing 12% methanol, 0.014 g/l EDTA and 0.1 mM sodium-1-octansulfonate pumped at a flow rate of 1 ml/min. All chromatography experiments were performed at 4°C and all solutions were kept on ice. The separations were achieved under isocratic conditions. The detector cell was operated at +0.8 V.

### Quantitative PCR

Total mRNA was extracted from neurons using the RNeasy Kit (Qiagen) followed by a reverse-transcription to obtain cDNA using the Maxima First Strand cDNA Synthesis Kit (Thermo scientific). Quantitative RT-PCR was performed with the Maxima SYBR^®^ Green/fluorescein qPCR Master Mix kit (ThermoFisher) on a LightCycler 480 (Roche Diagnostics). Quantification was performed using the deltadelta-CP (ΔΔCP) method (Livak and Schmittgen, 2001). All oligonucleotides used are summarized in Supplementary Table 1 along with the corresponding target genes.

### Quantification of TH-Positive Neurons

To determine the efficiency of differentiation into TH^+^ neurons, Poly-L-Ornithine/Laminin/Fibronectin-coated coverslips containing terminally differentiated iPSC- or NPC-derived neurons were fixed and stained using an anti-TH antibody and mounted using DAPI-containing mounting medium, as mentioned above. On each coverslip, five randomly chosen sections were imaged using a confocal microscope (Zeiss LSM710) together with a 40x objective. Cell counting and evaluation of the differentiation efficiency were performed manually. Six coverslips obtained from two independently differentiated cultures were evaluated for each iPSC- or NPC-derived culture. The total number of cells analyzed exceeded 1,000 cells.

### Electrophysiology

**FIGURE 1:**
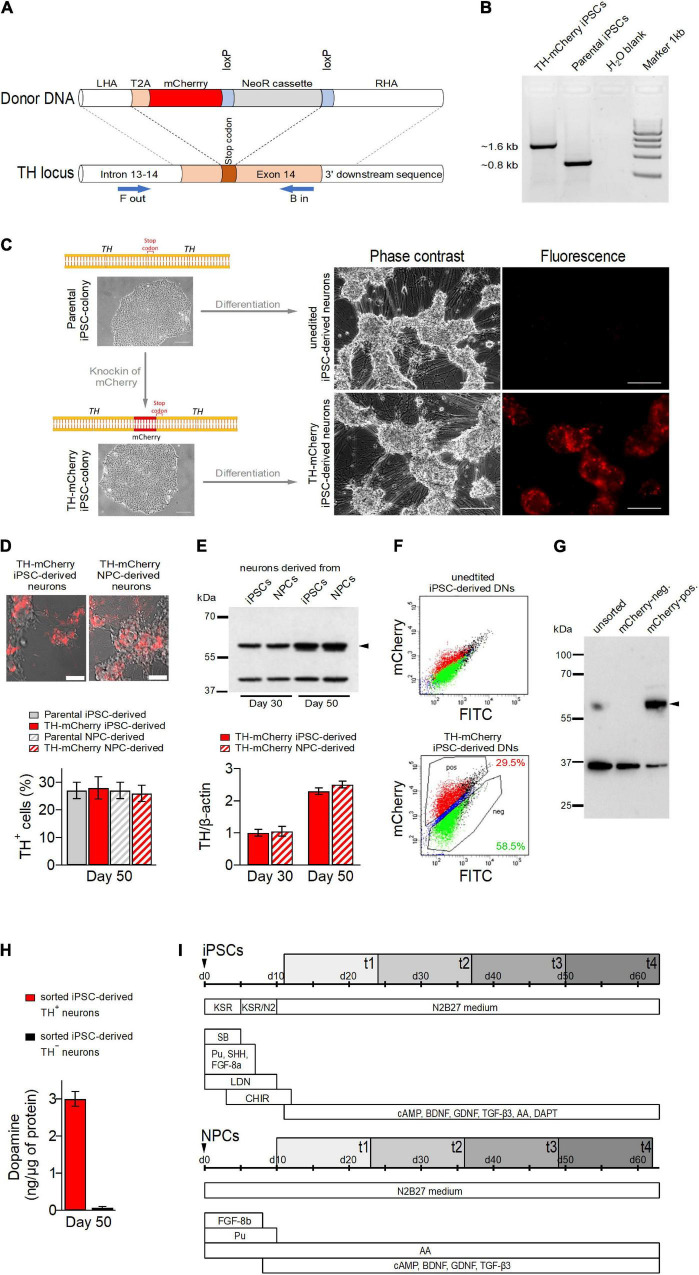
Generation of TH-mCherry reporter iPSC and NPC lines and associated dopaminergic neuronal cultures. **(A)** Scheme illustrating the recombination strategy employed for genome editing. Blue arrows represent primers used for the PCR screening procedure. **(B)** PCR analysis of edited (TH-mCherry iPSCs) and unedited (Parental iPSCs) iPSC lines confirming correct integration of the T2A-mCherry sequence in the TH locus of the parental iPSC line. **(C)** Phase contrast and associated fluorescence images of representative clusters of undifferentiated iPSC colonies (*left*) and terminally differentiated DN cultures (*right*), obtained from unedited (unedited iPSC-derived neurons) or edited (TH-mCherry iPSC-derived neurons) iPSCs (*left*). Scale bars: 100 μm. **(D)** Live-cell images of TH-mCherry reporter DN cultures derived from iPSCs or NPCs (*top*, scale bars: 50 μm) and analysis (*bottom*) of the percentage of TH-positive cells present in unedited and edited iPSC- and NPC-derived neuronal cultures, obtained from fixed samples immunostained with a TH-specific antibody. For each condition, a total of six replicates from two independent differentiations were evaluated. **(E)** Representative Western blot image (*top*) and associated densiometric analysis (*bottom*) of TH protein levels in 30- and 50-day-old iPSC- and NPC-derived dopaminergic neuronal cultures. β−Actin (42 kDa, *top*) served as loading control, bands corresponding to TH (60 kDa, *top*) are marked with an arrowhead. Bars represent means ± s.e.m. obtained from two independent experiments each. **(F)** Representative two-component density plots for FACS sorting of terminally differentiated parental (*top*) and TH-mCherry reporter iPSC cultures (*bottom*) using FITC- and mCherry-specific detection channels. The unspecific autofluorescence of unedited iPSC-derived neurons served as template to set the sorting gate for mCherry-negative cells and to define the gate corresponding to mCherry-positive cells. **(G)** Western blot analysis to evaluate TH protein (arrowhead) in unsorted, FACS-sorted mCherry-negative and mCherry-positive cells. GAPDH (36 kDa) served as loading control. **(H)** Intracellular dopamine levels of TH^+^ and TH^–^ iPSC-derived neurons, normalized to total protein. Bars represent means ± s.e.m. obtained from two independent experiments. **(I)** Timelines summarizing the two differentiation strategies used for the creation of iPSC- (top) and NPC-derived DNs. Electrophysiological analysis of cells was performed during an 8-week period (gray rectangles), t1-t4 specify the four timing groups.

Individual cells were subjected to whole-cell patch-clamp experiments 1–8 weeks after initiation of the neuronal differentiation process (Figure 1G). To analyze the time course of electrophysiological maturation of cells and to facilitate statistical analysis, data were clustered into 4 timing groups (t1-t4), each representing a period of 2 consecutive weeks (t1: weeks 1 and 2, t2: weeks 3 and 4, t3: weeks 5 and 6, t4: weeks 7 and 8). Voltage- and current-clamp data were acquired using an EPC-10 patch-clamp amplifier, operated by PatchMaster software (both HEKA Elektronik, Lambrecht, Germany). All recordings were performed at a constant temperature of 20 ± 0.5°C using a microincubation stage (ALA Scientific Instruments, Farmingdale, NY, United States), feedback-controlled by a PTC-10 temperature controller (NPI Electronic GmbH, Tamm, Germany). Recording pipettes with resistances of 2–4 MΩ were fabricated from borosilicate glass and coated with silicone elastomer (RTV 615, Momentive Performance Materials, Waterford, NY, United States) to reduce tip capacitance. Series resistance was corrected electronically up to 80% and all voltages were corrected off-line for the liquid junction potential (−7 mV). Only cells with an uncompensated series resistance < 20 MΩ were included in the analysis. Data were low-pass filtered at 2.87 kHz and digitized with a sampling interval of 50 μs. For voltage- and current-clamp recordings patch pipettes were filled with (in mM) 125 KCl, 8 NaCl, 1 CaCl_2_, 1 MgCl_2_, 0.4 Na_2_-GTP, 4 Mg-ATP, 10 EGTA and 10 HEPES (pH 7.3 with KOH). The bath solution contained in all experimental conditions (in mM) 120 NaCl, 3 KCl, 2.5 CaCl_2_, 1 MgCl_2_, 30 HEPES, 15 glucose (pH 7.4 with NaOH).

#### Voltage-Clamp Recordings

Sodium and potassium channel-specific currents were evoked from a holding potential of −107 mV with a two-step voltage protocol. Activation of mostly voltage-gated sodium channels was triggered with a series of 20 ms test depolarizations ranging from −87 to −7 mV in steps of 10 mV followed by a 140 ms recovery period at −107 mV. Subsequently, voltage-gated potassium channels were activated with a series of 80 ms test depolarizations ranging from −7 to 73 mV in steps of 10 mV. The repetition interval was 2 s. Leak and capacitive currents were recorded using a P/4 method with four leak pulses, each with 0.15 time the amplitude of the test pulse P. Summed and scaled leak traces were subtracted online using the leak pulse feature available in PatchMaster software. Sodium and potassium channel-specific peak current amplitudes were normalized to the cell membrane capacitance and are reported as current densities, *I(V)*/*C*_*m*_, where *I(V)* is the peak current amplitude and *C*_*m*_ the cell membrane capacitance.

The membrane input resistance, *R*_in_, was estimated from current responses to depolarizing voltage pulses to −87, −77, and −67 mV, from which leak currents were not subtracted. The holding potential was −107 mV. *R*_in_ was obtained as the inverse of the linear slope of the associated current-voltage relationship.

Inward post-synaptic currents (PSCs) were recorded at a holding potential of −77 mV for a period of 20 s. PSCs measured under these conditions are a superposition of spontaneous and network activity-driven events. Recordings were analyzed for the occurrence of PSCs using IgorPro software (WaveMetrics, Lake Oswego, OR, United States) and customized scripts. A threshold-crossing of the first derivative of the current responses was used as objective detection criterion for PSCs. The detection threshold was set to −4 × σ_d_*_*I*_*_/d_*_*t*_* where σ_d_*_*I*_*_/d_*_*t*_* is the standard deviation of the first derivative of the current responses. Detected events were verified by visual inspection and further analyzed regarding their peak amplitudes and frequencies.

#### Current-Clamp Recordings

Spontaneously fired action potentials were recorded for a period of 30 s at zero current injection. The detection threshold for action potentials was set to −20 mV. Individual action potentials fired during this period were further analyzed for their peak voltage.

To obtain evoked action potential responses, cells were clamped to −77 mV and repetitively stimulated with 2 s current injections ranging from 0 to 180 pA in steps of 20 pA, delivered at an interval of 3 s. Parameters characterizing evoked action potentials were obtained from the first action potential fired during the stimulation paradigm.

#### Data Analysis and Statistics

Data analysis was performed using FitMaster software (HEKA Elektronik, Lambrecht, Germany) and Igor Pro (WaveMetrics, Lake Oswego, OR, United States). Data points are presented as means ± s.e.m. (*n*) with *n* being the number of independent experimental replicates. Data sets were tested for statistical significance with an unpaired 2-tailed Mann–Whitney *U*-test for averaged data or Fisher’s exact test for proportions when appropriate.

## Results

### Generation of a TH-mCherry Reporter Induced Pluripotent Stem Cell Line Using CRISPR/Cas9-Mediated Genome Editing

To distinguish TH^+^ from TH^–^ cells in DN cultures and to facilitate the analysis of their specific electrophysiological properties, we generated TH reporter iPSCs by incorporating the open reading frame of the fluorescent protein mCherry into the TH locus of a human control iPSC line (Zanon et al., 2017) using a previously described strategy (Calatayud et al., 2019).

The iPSCs were co-transfected with a plasmid expressing guide RNA (gRNA), SpCas9, and a plasmid containing a T2A-mCherry fusion as donor (Figure 1A) followed by Neomycin selection for 7 days. Resistant clones were analyzed for correct integration of the donor DNA using a PCR-based assay (Figure 1B) and Sanger sequencing upon excision of the resistance cassette. The successfully edited iPSC line with correct biallelic integration of T2A-mCherry in the TH locus was expanded and analyzed for the presence of pluripotency markers. Control and edited iPSC lines showed positive expression of pluripotency markers OCT4, NANOG, TRA-1-60, and SSEA4 (Supplementary Figure 1A) and gene expression of NANOG, OCT4, GDF3, and SOX2 was elevated in both unedited and edited iPSCs compared to parental fibroblasts (Supplementary Figure 1B).

### Expression of mCherry Correlates With the Expression of TH in Differentiated Neuronal Cultures

To yield DN cultures, unedited and TH reporter iPSCs were differentiated toward dopaminergic SNpc neurons following two widely used protocols, i.e., the “midbrain floor plate protocol” (Kriks et al., 2011), and a protocol that utilizes iPSC-derived neuronal progenitor cells (NPCs) (Reinhardt et al., 2013). NPCs were obtained from both parental iPSCs and TH reporter iPSCs using small molecules (Reinhardt et al., 2013) and subsequently characterized by immunochemistry and qPCR. As shown in Supplementary Figures 1C,D, both unedited and TH reporter NPCs expressed comparable levels of NPC-specific markers Musashi 1, Nestin, and Pax6 which were also considerably higher compared to in iPSCs, demonstrating (a) the generation of NPCs was successful and (b) genetic editing did not affect the efficiency of conversion of iPSCs to NPCs.

Unedited and TH reporter iPSCs were differentiated for 50 days (Figure 1C) to yield DN cultures. Both cultures developed a comparable neuronal morphology. While red fluorescence was absent in DNs obtained from unedited iPSCs, it was observed in a subset of cells derived from TH-mCherry reporter iPSCs as early as 15 days after the differentiation process was triggered. Similar observations were made for NPC-derived TH reporter cultures. As revealed by live cell imaging after 50 days of differentiation, NPC-derived TH reporter cultures were also indistinguishable from iPSC-derived TH reporter cultures with respect to their morphological development (Figure 1D, *top*). Immunochemical analysis of fixed samples revealed comparable proportions of TH-immunoreactive cells in DN cultures obtained from both unedited and TH reporter iPSCs and NPCs (parental iPSC-derived neurons: 27 ± 3%, TH-mCherry iPSC-derived neurons: 28 ± 4%, parental NPC-derived neurons: 27 ± 3%, TH-mCherry NPC-derived neurons 26 ± 3%) further confirming that genetic editing did not interfere with the differentiation efficiency (Figure 1D, *bottom*). In addition, relative TH expression levels in TH-mCherry iPSC- and NSC-derived cultures were compared 30 and 50 days after the onset of differentiation using a Western blot assay. As shown in Figure 1E, both cultures expressed TH at comparable levels, which also increased equally in both cultures with increasing differentiation time.

To test whether mCherry expression overlaps with TH expression, DNs derived from TH reporter iPSCs using the “midbrain floor plate protocol” were sorted into mCherry-positive and mCherry-negative cell populations using fluorescence-activated cell sorting (FACS) (Figure 1F). DNs obtained from unedited iPSCs were used to define the sorting gates. Subsequently, whole protein was isolated from unsorted cells and from sorted mCherry-positive and mCherry-negative cell populations. As shown in Figure 1G, western blot analysis with a TH-specific antibody confirmed the presence of TH in unsorted cells and in the mCherry-positive cell population, while TH expression was not detected in mCherry-negative cells. The sorted cell populations were also analyzed for their ability to synthesize dopamine by means of HPLC-coupled electrochemical detection. As shown in Figure 1H, dopamine could be detected exclusively in the lysates of TH^+^ neurons, confirming the dopaminergic nature of this cell type. However, the dopamine metabolites DOPAC and HVA were not detected in the lysates, possibly because they were released from the cells during the separation procedure, or their amounts were below the detection limit of our HPLC system.

Taken together, this demonstrates that mCherry-mediated red fluorescence in TH reporter DNs is a reliable indicator of both endogenous TH expression and dopamine synthesis.

### Assessment of Ion Channel Function and Synaptic Activity in SNpc Neurons Derived From Neuronal Precursor Cells and Induced Pluripotent Stem Cells

Electrical excitability is a fundamental feature of mature neurons and requires the concerted activity of various types of ion channels. Especially voltage-gated ion channels, such as Na_V_ and K_V_ channels, are important in neurons because they trigger and shape action potentials. To assess the functional expression of these channels, we performed whole-cell voltage-clamp measurements on TH^–^ and TH^+^ NPC- and iPSC-derived neurons and analyzed Na_V_- and K_V_-specific current components in these cells at four different time points (t1-t4), each combining data from two consecutive weeks (t1: weeks 1–2, t2: weeks 3–4, t3: weeks 5–6, t4: weeks 7–8) after initiation of neuronal maturation (Figure 1I).

**FIGURE 2:**
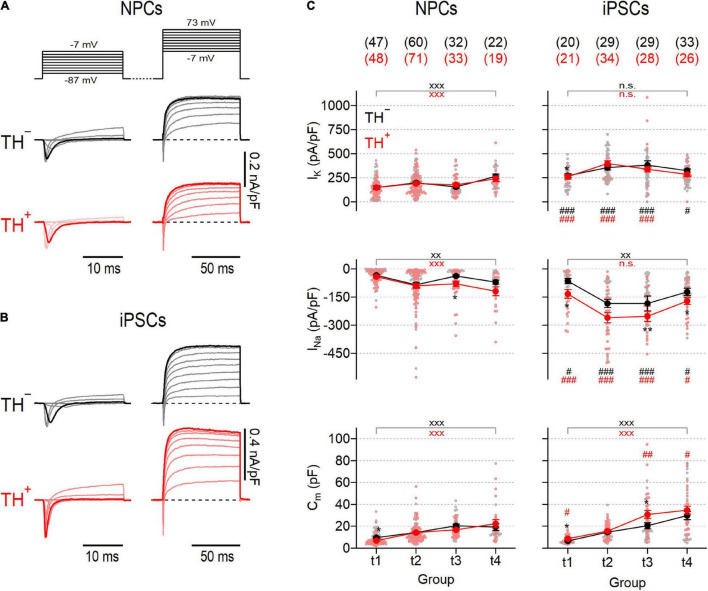
Activity of voltage-gated Na_V_ and K_V_ channels in NPC- and iPSC-derived dopaminergic reporter lines. Families of representative current traces obtained from NPC-derived **(A)** or iPSC-derived **(B)** neurons either positive (TH^+^, red) or negative (TH^–^, black) for the expression of TH in response to various step depolarizations (*top, **A***). 20-ms depolarizations ranging from –87 to –7 mV (*left*) were applied to elicit mostly Na_V_-specific fast activating and inactivating inward currents, whereas slow activating outward currents reflecting the activity of K_V_ channels were triggered with 80-ms depolarizations ranging from to –7 mV to 73 mV (*right*). Inward and outward current traces highlighted in bold correspond to test pulse voltages to –27 mV and 73 mV, respectively. Data in **(A,B)** were obtained at time point 2 (t2), i.e., 3–4 weeks after neuronal differentiation was initiated. **(C)** Mean peak current densities associated to K_V_ (*top*) or Na_V_ (*middle*) channels and mean cell membrane capacitances (*bottom*), obtained from current traces at 73 mV and –27 mV, respectively, as shown in **(A,B)** at the indicated time points (t1–4). Individual data points are shown as open circles, filled circles represent means ± s.e.m. with the number of experimental replicates, *n*, indicated in parentheses. Straight lines connect means for clarity. Significance between pairs of data was tested with a two-tailed Mann–Whitney *U*-test: **P < 0.01, *P < 0.05 for testing TH^–^ against TH^+^ neurons from the same line; ^xxx^*P* < 0.01, ^xx^*P* < 0.01 for testing data obtained at t1 against data obtained at t4; ^###^*P* < 0.001, ^##^*P* < 0.01, ^#^*P* < 0.05 for testing data obtained from NPCs against data obtained from iPSCs; n.s., not significant.

To obtain Na_V_− and K_V_-specific current responses, we used a double-pulse protocol as shown in Figure 2A. Na_V_-dependent inward currents were triggered with a first series of brief test pulses ranging from −87 to −7 mV followed by a second test pulse series, ranging from −7 to 73 mV, to activate K_V_-dependent outward currents. Since the voltage dependencies of inward and outward currents largely overlapped (Figures 2A,B and Supplementary Figure 2), further analysis of Na_V_-specific currents was restricted to a test pulse voltage of −27 mV where Na_V_ channels but barely any K_V_ channels are active. K_V_-specific currents were further analyzed at a voltage of 73 mV, which is close to the calculated Na^+^ reversal potential (68.4 mV) at which the contribution of Na_V_-dependent currents is expected to be neglectable. Both, NPC- and iPSC-derived neurons generated inward and outward currents with kinetics typical for Na_V_ and K_V_ channels, respectively (Figures 2A,B). The associated peak current densities in NPC-derived neurons increased gradually during differentiation without apparent differences between TH^–^ and TH^+^ cells (Figure 2C). Both current components were consistently larger in iPSC-derived neurons than in NPC-derived neurons and exhibited a biphasic development characterized by an increase of current densities from t1 to t2, a plateau phase between t2 and t3 and a decrease to almost initial levels at t4. K_V_-specific currents were not different between TH^–^ and TH^+^ iPSC-derived neurons, but Na_V_ channel-mediated currents were larger in the TH^+^ subpopulation at all-time points tested, being significant for groups at t1, t3 and t4 (Figure 2C). The cell membrane capacitance, a passive property that scales with the cell membrane surface area, increased significantly during differentiation in NPC- as well as in iPSC-derived neurons by factors of 4.0 and 4.6, respectively (both *P* < 0.001, Figure 2). As shown in Supplementary Figure 3, the membrane input resistance (*R*_in_) of NPC-derived TH^–^ neurons was independent of the time in differentiation but decreased by a factor of approximately 2 in corresponding TH^+^ cells from 3.9 ± 0.5 GΩ at t1 to 2.0 ± 0.3 GΩ at t4 (*P* < 0.05). In contrast, *R*_in_ of both iPSC-derived subpopulations decreased continuously from t1 to t3 before reaching a plateau at t4 (TH^–^: from 2.5 ± 0.3 GΩ at t1 to 1.5 ± 0.2 GΩ at t4, *P* < 0.01; TH^+^: from 3.5 ± 0.7 GΩ at t1 to 1.1 ± 0.2 GΩ at t4, *P* < 0.001). In neither cell line did *R*_in_ correlate with the expression of TH.

**FIGURE 3:**
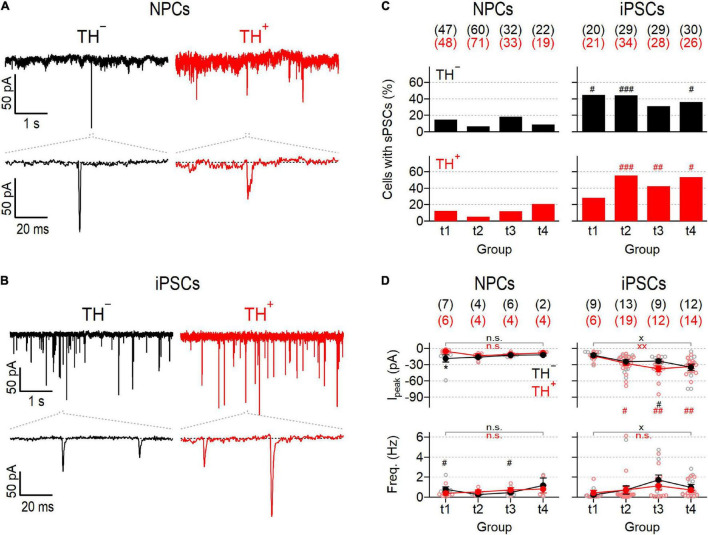
Different synaptic activity in NPC- and iPSC-derived dopaminergic reporter cultures. **(A)** Representative post-synaptic current responses (PSCs) at time point 2 (t2) from TH^–^ (*left*) and TH^+^ (*right*) NPC-derived neurons at a membrane potential of –77 mV. Bottom traces are enlarged views (100 ms) of the regions specified with dashed lines. **(B)** Identical recordings as in **(A)**, obtained from TH^–^ (*left*) and TH^+^ (*right*) iPSC-derived neurons at t2. **(C)** Bar graphs showing percentages of NPC- (*left*) and iPSC-derived (*right*) TH^–^ (*top*, black) and TH^+^ (*bottom*, red) neurons that showed PSCs as shown in **(A,B)**, analyzed at the specified time points (t1–4). Statistic indicators refer to Fisher’s exact test performed for each time point between data obtained from NPC- and iPSC-derived neurons:^ ###^*P* < 0.001, ^##^*P* < 0.01, ^#^*P* < 0.05. **(D)** Mean peak current amplitudes (*top*) and frequencies (*bottom*) of PSCs, obtained from NPC- (*left*) or iPSC-derived (*right*) TH^–^ or TH^+^ neurons at different time points during maturation. Filled circles are means ± s.e.m. with the numbers of experimental replicates, *n*, indicated in parentheses. Straight lines connect means for clarity. Individual data points are shown as open circles. Statistical significance between data pairs was analyzed with a two-tailed Mann–Whitney *U*-test: *P < 0.05 for testing TH^–^ against TH^+^ of the same line; ^xx^*P* < 0.01, ^x^*P* < 0.05 for comparing data obtained at t1 with data obtained at t4; ^##^*P* < 0.01, ^#^*P* < 0.05 for testing data derived from iPSCs against data derived from NPCs.

To make an inference on the development of the synaptic apparatus in NPC- and iPSC-derived neuronal cultures, post-synaptic currents (PSCs) were recorded in the absence of specific inhibitors (Figures 3A,B). Post-synaptic events detected under these conditions reflect network activity driven by action potential-dependent and -independent mechanisms (Pinheiro and Mulle, 2008). Independent of the time in differentiation, PSCs were evident in less than 20% of NPC-derived TH^–^ and TH^+^ neurons, suggesting a low level of synaptic activity in this culture (Figure 3C). Mean frequencies and amplitudes of the events varied marginally during differentiation between 0.26 ± 0.05 Hz and 1.18 ± 0.76 Hz and between −5.5 ± 1.2 pA and −18.6 ± 6.5 pA for TH^–^ and TH^+^ cells, respectively, and were virtually independent of TH expression (Figure 3D). By contrast, the proportions of iPSC-derived neurons exhibiting PSCs varied between 28.6% and 55.9% during differentiation, demonstrating an overall higher degree of synaptic activity compared to neurons obtained from NPCs. Although variable, the percentage of iPSC-derived cells capable of generating PSCs was independent of both, TH expression and time in differentiation (Figure 3C). The mean frequencies of events were comparable to those observed in neurons obtained from NPCs and showed no obvious time-dependence, either. However, the mean amplitudes of the events increased gradually in both iPSC-derived subpopulations over time (Figure 3D): At an early phase of differentiation at time point t1, they were almost indistinguishable from the amplitudes measured in NPC-derived cells, but by time point t4 they had more than doubled (t1: −12.7 ± 2.6 pA and −13.8 ± 3.5 pA, t4: −34.7 ± 6.1 pA and −33.6 ± 3.5 pA for TH^–^ and TH^+^, respectively).

In summary, the direct differentiation of iPSCs to DNs yielded a neuronal population that contained more functional Na_V_ and K_V_ channels and displayed a higher degree of synaptic activity compared to DNs generated using an indirect differentiation strategy involving neural precursors.

### Action Potential Characteristics in Neuronal Precursor Cell- and Induced Pluripotent Stem Cell-Derived TH Reporter Cultures

**FIGURE 4:**
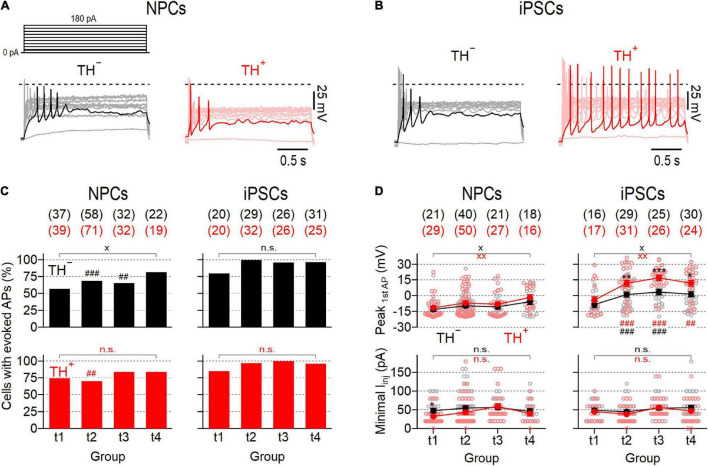
Evolution of evoked action potential responses in NPC- and iPSC-derived dopaminergic cultures. **(A)** Representative voltage responses (*bottom*) of TH^–^ (*left*) and TH^+^ (*right*) NPC-derived neurons at time point 3 (t3) to 2 s current injections ranging from 0 to 180 pA (*top*). The 20 pA stimulation step is shown bold in the pulse protocol, corresponding voltage responses are highlighted in black (TH^–^) and red (TH^+^). **(B)** Identical recordings as in **(A)**, from TH^–^ and TH^+^ iPSC-derived neurons at t3. **(C)** Percentage of NPC- (*left*) and iPSC-derived (*right*) TH^–^ (*top*, black) and TH^+^ (*bottom*, red) neurons that responded to 2 s current injections of up to 180 pA with one or more APs, analyzed at specified time points. **(D)** Mean minimal current (*bottom*) required to evoke APs as shown in **(A,B)** and associated mean peak voltages (*top*) of the first AP fired, analyzed at indicated time points. Filled circles represent means ± s.e.m, connected with straight lines for clarity. Open circles indicate data obtained from individual cells. The numbers of cells analyzed in **(C,D)** are provided in parentheses. Statistical indicators in **(C,D)** refer to Fisher’s exact test or a Mann–Whitney *U*-test, respectively: ***P < 0.001, **P < 0.01, *P < 0.05 for testing TH^–^ against TH^+^ neurons of the same line; ^xx^*P* < 0.01, ^x^*P* < 0.05, n.s. for comparing data obtained at t1 with data from t4; ^###^*P* < 0.001, ^##^*P* < 0.01 for testing data obtained with iPSC-derived neurons against data from NPC-derived neurons.

To further characterize functional maturation of NPC- and iPSC-derived neuronal DN cultures, we performed whole-cell current-clamp recordings on individual TH^–^ and TH^+^ neurons from both lines at different time points after triggering dopaminergic differentiation and analyzed the ability of the cells to generate action potentials. To evoke responses, cells were kept at −77 mV and stimulated with escalating current injections ranging from 0 pA to 180 pA. Using this stimulation protocol one or more action potentials were triggered in 56.8–84.4% of NPC-derived neurons and in 80–100% of iPSC-derived neurons (Figures 4A–C). Independent of the amplitude of current injected, the time point in differentiation, and the differentiation protocol used, TH^–^ cells rarely fired more than one action potential in response to a current stimulus (Supplementary Figure 4). In contrast, in TH^+^ cells, the firing of evoked action potentials evolved throughout the differentiation period and was dependent on the differentiation protocol applied. Whereas the mean firing frequencies of NPC-derived TH^+^ cells increased above those of corresponding TH^–^ cells particularly in the last phase of differentiation (t4), the firing rates of iPSC-derived TH^+^ cells increased already from t2 and reached a maximum of 4.0 Hz at t3.

In both cultures the proportions of cells firing were independent of TH expression and varied only marginally between time points, except for the TH^–^ subpopulation of NPC-derived neurons, which showed a small yet significant increase of the proportion of excitable cells from 56.8% at t1 to 81.8% at t4 (*P* < 0.05). The minimum current amplitude required to initiate an action potential response was virtually identical in NPC- and iPSC-derived cultures among the entire differentiation period and did also not correlate with TH expression (Figure 4D, bottom). Analysis of the peak voltage of the first action potential fired during the stimulation paradigm, *V*_peak_, revealed significant differences between the two neuronal cell lines: While in both NPC-derived subpopulations *V*_peak_ did not exceed 0 mV at all-time points tested, it reached values above 0 mV in both iPSC-derived subpopulations already from time point t2, indicating that neuronal maturation was more efficient in the iPSC-derived culture. Moreover, *V*_peak_ was consistently higher in the TH^+^ subpopulation of iPSC-derived neurons compared to their TH^–^ counterparts, suggesting that electrophysiological maturation of TH^+^ cells was preferred in this culture.

**FIGURE 5:**
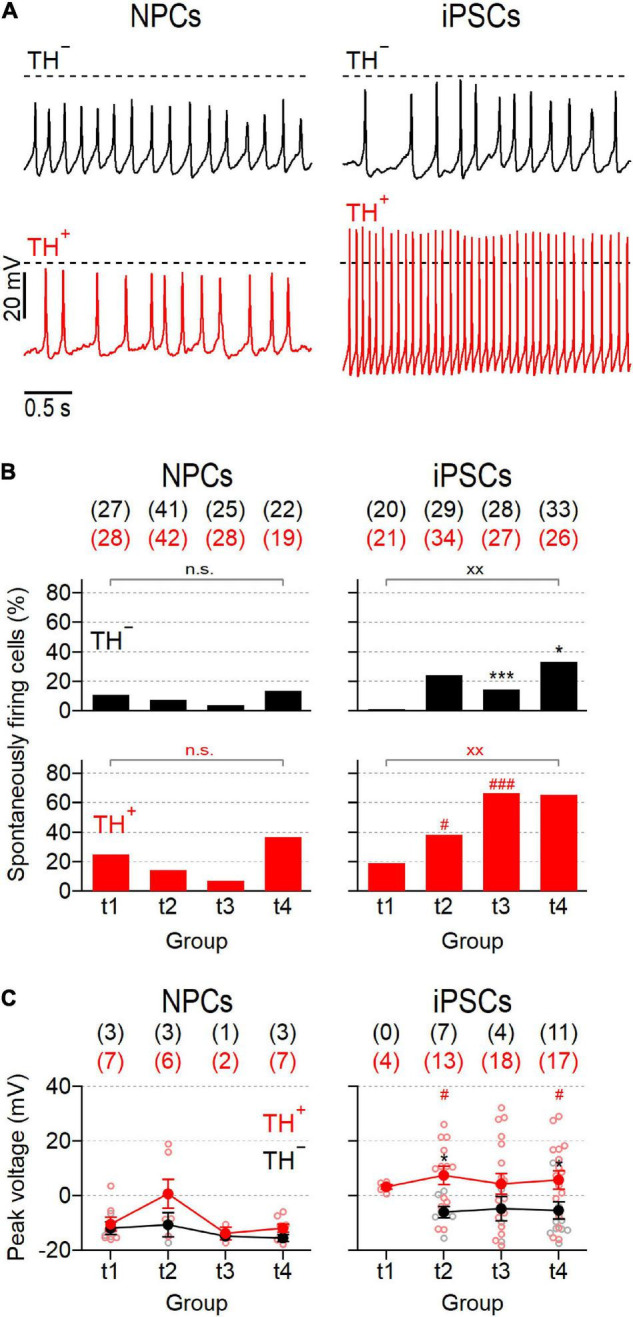
NPC- and iPSC-derived dopaminergic cultures contain different proportions of spontaneously firing neurons. **(A)** Representative action potential recordings obtained at time point 2 (t2) from NPC- (*left*) and iPSC-derived (*right*) neurons that were negative (TH^–^, *top*) or positive (TH^+^, *bottom*) for the expression of TH, in response to zero current injection. Dashed lines indicate a membrane potential of 0 mV. **(B)** Percentage of NPC- and iPSC-derived TH^–^ (*top*) and TH^+^ (*bottom*) neurons that fired spontaneous action potentials as shown in **(A)**, analyzed at different time points during maturation. For each condition, the total number of cells analyzed, *n*, is provided in parentheses. **(C)** Average peak voltages (filled circles) of spontaneously fired action potentials as shown in **(A)**, analyzed at specified time points. Data points are means ± s.e.m. with the numbers of cells analyzed, *n*, given in parentheses. Straight lines connect data for clarity. Open circles represent data obtained from individual cells. Statistical indicators in **(B,C)** refer to a Fisher’s exact test and a Mann–Whitney *U*-test, respectively: ***P < 0.001, *P < 0.05 for testing TH^–^ against TH^+^ neurons of the same line;^xx^ *P* < 0.01 for comparing data obtained at t1 with data obtained at t4;^###^ *P* < 0.001, ^#^*P* < 0.05 for testing data obtained from NPC-derived neurons against data obtained from iPSC-derived neurons; n.s. not significant.

Since spontaneous activity is a hallmark feature of mature SNpc neurons (Guzman et al., 2009), we also analyzed the ability of the cells to generate action potentials in the absence of a current stimulus. Representative recordings obtained from both subpopulations of NPC- and iPSC-derived neurons are shown in Figure 5A. The proportions of spontaneously firing cells were generally low in NPC-derived neurons ranging from 4.0 to 13.6% and from 7.1 to 36.8% for TH^–^ and TH^+^ cells, respectively (Figure 5B). Although spontaneous firing was more frequently detected in TH^+^ cells than in TH^–^ cells at all-time points tested, these differences did not reach the level of significance. Independent of the time in differentiation and the expression of TH, the mean peak voltages of spontaneous action potentials did not exceed 0 mV, except for TH^+^ cells at t2, which fired trains of action potentials with a mean peak voltage of 0.7 ± 5.2 mV (Figure 5C). As sown in Figure 5B, the proportions of spontaneously active cells increased in the iPSC-derived culture continuously over time and exhibited noticeable differences between the TH^–^ and TH^+^ subpopulations, particularly in late phases of differentiation: In TH^–^ neurons, spontaneous firing occurred first at time point t2 (24.1%) and increased only slightly until t4 (33.3%). By contrast, the proportion of spontaneously active TH^+^ cells increased more rapidly as the culture matured, reaching significantly higher levels at t3 (66,7%, *P* < 0.001 vs. TH^–^) and t4 (65.4%, *P* < 0.05 vs. TH^–^). In addition, the average peak voltages of spontaneous action potentials were consistently higher in the iPSC-derived TH^+^ cell population compared to in the TH^–^ subpopulation (Figures 5A,C): While TH^–^ cells fired—at all-time points tested—trains of spontaneous action potentials with a mean peak voltage below 0 mV, the TH^+^ cells generated action potentials with an average peak voltage above 0 mV, being significant for groups at t2 and t4 (both *P* < 0.05). To identify possible mechanisms underlying the different electrophysiological properties of TH^+^ and TH^–^ cells, both neuronal subpopulations were isolated from the iPSC-derived culture at day 50 of differentiation (corresponding to t4) by FACS and subsequently analyzed for the mRNA levels of the neuronal progenitor markers Pax6, SOX2, and ASCL1. As shown in Supplementary Figure 5, TH^–^ cells expressed Pax6 and SOX2 at higher levels than TH^+^ neurons (*P* < 0.05), whereas ASCL1 was expressed almost equally in both subpopulations.

Thus, mature action potentials developed preferentially in TH^+^ iPSC-derived cells, whereas the associated TH^–^ cells as well as both NPC-derived subpopulations were characterized by electrophysiological features characteristic of premature neuronal precursors.

## Discussion

Human iPSCs are widely used to model physiological and pathophysiological conditions because they can be differentiated into a variety of cell types (Takahashi et al., 2007; Yu et al., 2007; Dimos et al., 2008; Ebert et al., 2009; Soldner et al., 2009; Guimaraes et al., 2018) including SNpc-associated DNs, which play a major role in the pathogenesis of PD. Several procedures have been developed that enable the differentiation of DNs from iPSCs, all with specific advantages and disadvantages (Chambers et al., 2009; Kirkeby et al., 2012; Xi et al., 2012; Doi et al., 2014). However, all differentiation strategies available to date give rise to largely inhomogeneous DN cultures containing variable proportions of neurons capable of synthesizing dopamine (TH^+^) and morphologically indistinguishable non-dopaminergic (TH^–^) cells which lack TH expression. This variability makes the targeted analysis of the PD-relevant TH^+^ subpopulation difficult, especially when functional assays are involved, which require living neurons (Calatayud et al., 2019; Laperle et al., 2020).

To overcome this issue, we adapted an established strategy (Xia et al., 2017; Calatayud et al., 2019; Hong and Daadi, 2019; Uberbacher et al., 2019) and engineered an mCherry-based TH reporter iPSC line, which enables visual identification of cells expressing TH, the rate-limiting enzyme in dopamine synthesis. The subsequent differentiation into DN cultures was performed according to two well-established procedures to evaluate if and how protocol-specific conditions affect the electrophysiological characteristics of DNs. First, we applied a protocol that converts iPSCs into expandable and easy-to-handle NPCs before differentiating them to DNs (Reinhardt et al., 2013). In a second set of experiments, we followed a “midbrain floor plate protocol” to yield DNs directly from iPSCs (Kriks et al., 2011).

Both NPC- and iPSC-derived DN cultures were morphologically indistinguishable and contained comparable proportions of TH^+^ neurons, ranging between 26 and 28%, which is compatible with most previous studies reporting yields of between 3 and 30% TH^+^ cells relative to all cells (Sundberg et al., 2013; Ishikawa et al., 2016; Awad et al., 2017; Beevers et al., 2017; Romero-Moya et al., 2017; Sheng et al., 2017). Variable yields of TH^+^ cells at low levels are a known limitation of all differentiation strategies available to date, largely attributed to the asynchronous maturation of cells due to a stochastic component inherent in the differentiation process that ultimately leads to unequal responses of iPSCs to morphogens (Antonov and Novosadova, 2021). In addition, iPSCs may be epigenetically unique, such that their potential to differentiate into a specific sublinage may be predetermined (Hu et al., 2010). Laboratory-specific handling of the cells is also considered to affect the conversion efficiency (Mahajani et al., 2019).

However, approximately 10 days after dopaminergic differentiation was initiated, cells from both cultures started to cluster and adopted a neuronal morphology. In both cultures, the morphological development was accompanied by a continuous increase of the cells’ membrane capacitance, indicating that cell surface area and thus cell size increased during differentiation as it is expected for developing cultures (Golowasch et al., 2009). Moreover, the input resistance (*R*_in_), a passive membrane property inversely correlated with cell size and open channel density, was in both cultures independent of TH expression but decreased with increasing differentiation time in both iPSC-derived subpopulations, which is consistent with previous studies focusing on developing human iPSC-derived DNs (Pre et al., 2014) or postnatal DNs obtained from rats (Dufour et al., 2014). Further evidence of morphological and functional maturation is that *R*_in_ levels at late stages of differentiation (t3-t4) were comparable to those previously reported for terminally differentiated iPSC-derived cortical linage neurons including DNs (Stanslowsky et al., 2014; Gunhanlar et al., 2018).

Na_V_− and K_V_-dependent current densities, both of which are essential for action potential formation, increased during differentiation in cells from both cultures (Figure 2) further confirming that both differentiation strategies yielded cells with developing neuronal characteristics. Furthermore, measured mean current densities, as well as their evolution during maturation, are consistent with previous studies examining the electrophysiological properties of human non-reporter NPC- and iPSC-derived DNs (Reinhardt et al., 2013; Pre et al., 2014; Stanslowsky et al., 2014). Although Na^+^ and K^+^ currents increased during maturation in cells from both cultures, they were always larger in iPSC-derived neurons compared to neurons obtained from NPCs. In addition, the TH^+^ subpopulation of iPSC-derived neurons generated larger Na_V_-dependent current amplitudes than the corresponding TH^–^ neurons, a property that was not observed in the NPC-derived reporter culture (Figure 2).

The ability of neurons to communicate with each other *via* synaptic contacts is a fundamental property of mature neural networks that is continuously and dynamically regulated (Glasgow et al., 2019). Synaptogenesis requires the coordinated interplay of various cellular processes, including those that control neurotransmitter release and postsynaptic recognition, and is therefore considered one of the signature endpoints of neurogenesis (Bradford and McNutt, 2015). Here we used a single-cell patch-clamp approach to analyze PSCs which are triggered by both pre- and postsynaptic processes and thus allow to make inferences on the synaptic activity of individual cells. In both DN cultures, the proportions of PSC-positive neurons were independent of TH expression and varied only marginally during differentiation. However, the proportions of PSC-generating neurons, and thus the overall synaptic activity, were on average more than three times higher in the iPSC-derived culture compared to in the NPC-derived culture (Figure 3C). In addition, only iPSC-derived neurons showed a progressive increase of PSC amplitudes, suggesting that the synaptic apparatus of these cells matured over time. The frequencies of events varied only marginally between time points t1 and t4, ranging from 0.3 to 1.7 Hz in both cultures. Together, these results are both qualitatively and quantitatively comparable to previous reports on NPC- and iPSC-derived SNpc neurons (Reinhardt et al., 2013; Pre et al., 2014; Stanslowsky et al., 2014; Gunhanlar et al., 2018).

The action potential characteristics of NPC- and iPSC-derived DNs also developed differently. Already 3–4 weeks after the differentiation process was triggered (t2), evoked and spontaneous action potentials were more frequently detected in iPSC-derived neurons and displayed more mature characteristics compared to the responses obtained from NPC-derived neurons (Figures 4, 5). Considering that the two differentiation protocols differ significantly in terms of the morphogens used and the timing of their application, and that both factors are crucially important for cell conversion, the different electrophysiological properties of NPC- and iPSC-derived neurons are not unexpected. For example, the combination of immediate specification of early neuronal progenitors by SHH/FGF8 and simultaneous activation of the Wnt signaling pathway by CHIR99021 as used in the “floor plate protocol” (Figure 1I) is considered to be particularly effective for obtaining DNs with a midbrain phenotype (Kriks et al., 2011; Weykopf et al., 2019; Antonov and Novosadova, 2021). Moreover, the presence of the Notch pathway antagonist DAPT may also have facilitated the electrophysiological maturation of iPSC-derived neurons, as it has been shown to promote the differentiation and maturation of derived neurons (Abranches et al., 2009; Borghese et al., 2010). In addition, the pluripotency of NPCs, and thus their ability to differentiate into mature neurons, appears to be negatively correlated with the number of cell passages they have experienced (Bardy et al., 2016).

However, the most striking observation was that the ability to generate action potentials with mature characteristics correlated in iPSC-derived neurons with the expression of TH while such a correlation was not seen in the NPC-derived DN culture: Throughout the 8-week differentiation period (t1–4), the action potential peak voltages of the iPSC-derived TH^+^ subpopulation were above 0 mV and significantly higher compared to the responses of TH^–^ cells. In addition, the proportions of spontaneously firing TH^+^ cells increased faster over time and were significantly larger compared to the proportions of spontaneously active TH^–^ neurons (Figure 5) particularly during late phases of differentiation. Together, these results demonstrate that of the two differentiation strategies evaluated in this work, the “midbrain floor plate protocol” (Kriks et al., 2011) yielded higher numbers of electrophysiologically mature neurons, most of which also expressed TH.

The reasons why TH^–^ and TH^+^ neurons matured differently in this culture cannot be inferred from the data shown here, but this finding is compatible with a previous study in which the transcriptome of TH^–^ and TH^+^ subpopulations of an iPSC-derived TH reporter line was analyzed (Xia et al., 2017). This work demonstrated that TH^+^ cells expressed genes with neuron-specific functions at higher levels than TH^–^ cells, which in contrast expressed elevated levels of cell cycle-associated genes suggesting the fraction of mature neurons was higher in the TH^+^ cell population whereas the TH^–^ subpopulation consisted mainly of undifferentiated neural precursors. In line with this interpretation, they found floor plate neuronal markers such as TH, LMX1B, ERBB3, NR4A2, and DDC enriched in TH^+^ cells, while TH^–^ cells were positive for neural stem cell markers such as Pax6, SOX2, ASCL1, and ZIC5TH and the non-midbrain floor plate markers HOXB1 and GLI2. Consistent with these data, we also found Pax6 and SOX2 enriched in TH^–^ cells. In another study it has been shown that TH^+^ and TH^–^ subpopulations of a TH reporter culture developed spontaneous calcium transients with distinguishable characteristics when analyzed in a Fluo-4AM-based imaging assay (Calatayud et al., 2019). This study demonstrated that amplitudes of spontaneous calcium signals were larger in TH^–^ neurons compared to TH^+^ cells, suggesting differences between the two neuronal subpopulations with respect to their electrical activity. However, although intracellular calcium signals are considered a proxy for spike activity (Kerr et al., 2005; Tian et al., 2009; Kwan and Dan, 2012; Chen et al., 2013), it is difficult to draw quantitative conclusions about cellular excitability solely based on calcium signals because elevated intracellular calcium levels can also be a consequence of suprathreshold depolarizations which do not trigger action potentials (Ali and Kwan, 2020).

In this study, we used single-cell electrophysiology as a direct measure of neuronal activity to compare for the first time the electrophysiological maturation of iPSC- and NPC-derived TH reporter DNs over a period of 8 weeks during differentiation. We demonstrated that although both differentiation strategies yielded basically identical proportions of TH^+^ cells with neuronal morphology, the fraction of electrophysiologically mature TH^+^ DNs was significantly larger in the iPSC-derived culture than in the NPC-derived culture suggesting that the direct differentiation of iPSCs to DNs using the “midbrain floor plate protocol” (Kriks et al., 2011) has the higher conversion efficiency to mature TH^+^ DNs, which represent the most vulnerable cell type in PD. However, given that NPC- and iPSC-derived cultures contained identical proportions of TH^+^ cells and expressed TH at comparable levels, it is evident that TH expression, although an important maturation marker for DNs, is not a sufficient indicator of the cells’ electrophysiological phenotype. Because excitability is an important physiological feature of mature neurons, electrophysiological properties of DNs derived from iPSCs should consequently be considered to monitor and evaluate the maturation process. Furthermore, the data support the notion that TH reporter DNs are a valuable tool for basic and translational PD research as they provide a means to restrict the analysis of cellular excitability and their PD-associated modulation to TH^+^ DNs, which is expected to reduce variability in associated data sets.

## Data Availability Statement

The original contributions presented in the study are included in the article/Supplementary Material, further inquiries can be directed to the corresponding author/s.

## Author Contributions

AR and EL designed the study. DV, FV, BM, and A-KH performed experiments. AR, DV, CN, CK, and EL analyzed and interpreted the data. AR, CN, CK, and EL wrote the manuscript with input from DV, FV, BM, and A-KH. All authors contributed to the article and approved the submitted version.

## Conflict of Interest

CK serves as medical advisor to Centogene for genetic testing reports in the fields of movement disorders and dementia, excluding Parkinson’s disease. The remaining authors declare that the research was conducted in the absence of any commercial or financial relationships that could be construed as a potential conflict of interest.

## Publisher’s Note

All claims expressed in this article are solely those of the authors and do not necessarily represent those of their affiliated organizations, or those of the publisher, the editors and the reviewers. Any product that may be evaluated in this article, or claim that may be made by its manufacturer, is not guaranteed or endorsed by the publisher.

## Footnotes

1
http://n2t.net/addgene:58778

2
http://n2t.net/addgene:13775
