# Supplementary material for: Electrophysiological Properties of Induced Pluripotent Stem Cell-Derived Midbrain Dopaminergic Neurons Correlate With Expression of Tyrosine Hydroxylase
Source: Front Cell Neurosci. 2022 Mar 23;16:817198. doi: 10.3389/fncel.2022.817198 (PMC8983830; doi:10.3389/fncel.2022.817198)
Supplement: Supplementary file 1 [file Data_Sheet_1.docx]

Supplementary Material

# Supplementary Methods

## Generation of neuronal precursor cells using small molecules

NPCs were generated as described previously (Reinhardt *et al.* 2013) with minor modifications to the protocol. In brief, iPSCs were disaggregated into single cells using Accutase (Stemcell) and plated in AggreWell 400 plates in mTeSR1 (both Stemcell), supplemented with ROCK inhibitor Y-27632 (EMD Biosciences). On the next day, iPSC aggregates were transferred onto an ultra-low attachment plate (Corning) containing EB medium (Knockout-DMEM with 20% Knockout-serum, 1% Penicillin/Streptomycin, 1% L-Glutamine, and 1% Non-essential amino acids (all from Thermo Fisher)) supplemented with CHIR99021 (Stemgent), SB431542 (SB, Tocris Bioscience), Dorsomorphin (Sigma) and Purmorphamine (Stemgent) and incubated for two days. On day three, EB medium was replaced with N2B27 Medium (DMEM/F12 and Neurobasal Medium 1:1, 1% Penicillin/Streptomicin, 1% L-Glutamine, 0.5% N-2 Supplement, and 1% B-27™ Supplement minus Vitamin A (all from Thermo Fisher)) supplemented with CHIR99021 (Stemgent), SB431542 (SB, Tocris Bioscience), Dorsomorphin (Sigma) and Purmorphamine (Stemgent). On day five, the medium was replaced with N2B27 medium supplemented with CHIR99021 (Stemgent), Purmorphamine (Stemgent), and ascorbic acid (Sigma). On day seven, cell aggregates were dissociated into single cells using Accutase (Stemcell) and transferred onto Matrigel (BD)-coated 6-well plates in N2B27 medium supplemented with CHIR99021 (Stemgent), Purmorphamine (Stemgent), and ascorbic acid (Sigma). Cells were passaged every 5-7 days until they formed colonies characteristic for NPCs which occurred usually after 3-5 passages.

## Generation of midbrain dopaminergic neurons

### Differentiation of iPSC into dopaminergic neurons

The direct differentiation of iPSCs into dopaminergic neurons was conducted as described previously (Kriks *et al.* 2011). In brief, iPSC colonies were disaggregated into single cells and plated onto Matrigel (BD)-coated dishes in mTeSR1 (Stemcell), supplemented with ROCK inhibitor Y-27632 (EMD Biosciences). Differentiation was started by adding knockout serum replacement (KSR) medium (Knockout DMEM with 15% knockout serum replacement (KSR), 2 mM L-glutamine (Gibco), 10 µM beta-mercaptoethanol, 0.1 mM MEM-NEAA (all from Thermo Fischer)) supplemented with SMAD pathway inhibitors SB431542 (SB, Tocris Bioscience) and LDN-193189 (LDN, Stemgent). On days 1 to 5, KSR medium was supplemented with SB, LDN, recombinant human sonic hedgehog (SHH, R&D System), recombinant human FGF-8a (R&D System) and Purmorphamine (Pu, Stemgent). The Wnt pathway activator molecule CHIR99021 (Stemgent) was included from days 3 to 12. During days 6–10 of differentiation, increasing amounts of Neurobasal Medium (Life Technologies) supplemented with SM1 (StemCell Technologies) was added to the KSR medium (25%, 50%, 75%) and upon day 7, SHH, FGF-8a, and Pu were withdrawn. On day 11, dopaminergic differentiation was initiated by adding recombinant human BDNF (Peprotech), recombinant human GDNF (Peprotech), ascorbic acid (Sigma), recombinant human TGF-β3 (Peprotech), cyclic-AMP (EnzoLifescience) and DAPT (Tocris, 10 µM). On day 20 of differentiation, cells were disaggregated into single cells and plated onto coverslips coated with poly-L-ornithine (Sigma), fibronectin (Sigma), and laminin (Sigma) and neuronal precursors were terminally differentiated for a period of 8 weeks.

### Differentiation of NPCs into dopaminergic neurons

The differentiation of NPCs into dopaminergic neurons was conducted as described previously (Reinhardt *et al.* 2013) with minor modifications. In brief, NPC colonies were disaggregated into single cells and plated onto Matrigel (BD)-coated dishes in N2B27 medium supplemented with recombinant human FGF-8b (R&D System), Purmorphamine (Pu, Stemgent), and ascorbic acid (Sigma). On day eight, FGF-8b was withdrawn. On day 10, cells were disaggregated into single cells and plated onto coverslips coated with poly-L-ornithine (Sigma), fibronectin (Sigma), and laminin (Sigma) and maintained in N2B27 medium supplemented with recombinant human BDNF (Peprotech), recombinant human GDNF (Peprotech), ascorbic acid (Sigma), recombinant human TGF-β3 (Peprotech), cyclic-AMP (cAMP, EnzoLifescience) for additional 20 days.

#
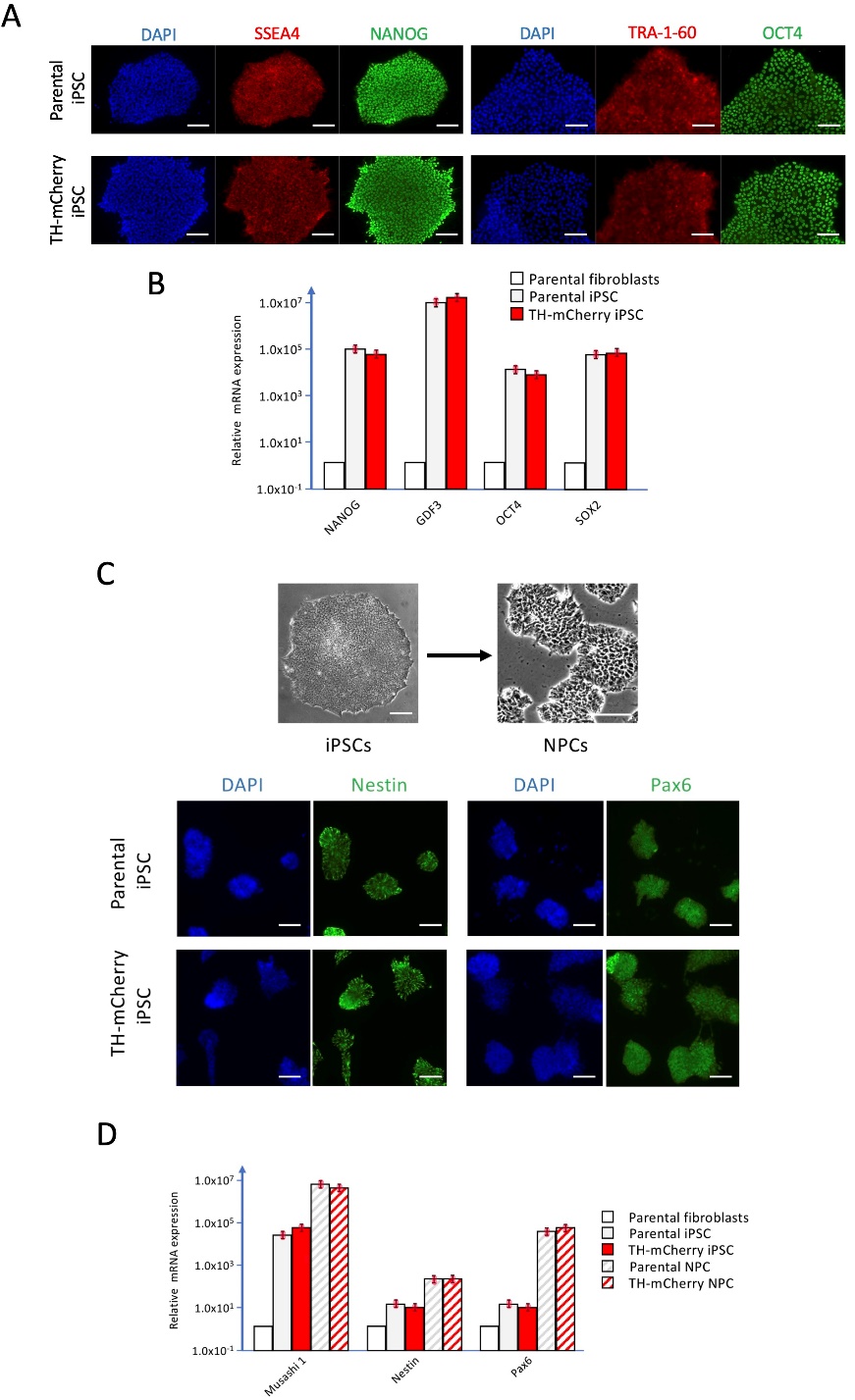
Supplementary Figures

**Supplementary Figure 1.** Characterization of the TH-mCherry reporter iPSC and NPC lines. **(A)** Immunofluorescence analysis of the parental and TH-mCherry reporter iPSCs showing the positive expression of pluripotency markers SSEA4, NANOG, TRA-1-60, and OCT4 (scale bars: 100 µm). **(B)** mRNA expression analysis of pluripotency markers NANOG, GDF3, OCT4, and Sox2 in the parental fibroblast line, parental iPSCs, and TH-mCherry reporter iPSCs. **(C)** Brightfield images illustrating different morphologies of iPSCs and iPSC-derived NPCs (*top*). As revealed by immunofluorescence staining (*bottom*), NPCs generated from both unedited (Parental iPSC) and edited (TH-mCherry iPSC) iPSCs were positive for NPC-specific markers Nestin and Pax6. Scale bars indicate 100 µm. **(D)** mRNA expression profile of NPC-specific markers Musashi 1, Nestin and Pax6 obtained from the cultures used in *C*. mRNA expression profiles in *B* and *D* are means of two independent measurements.

**
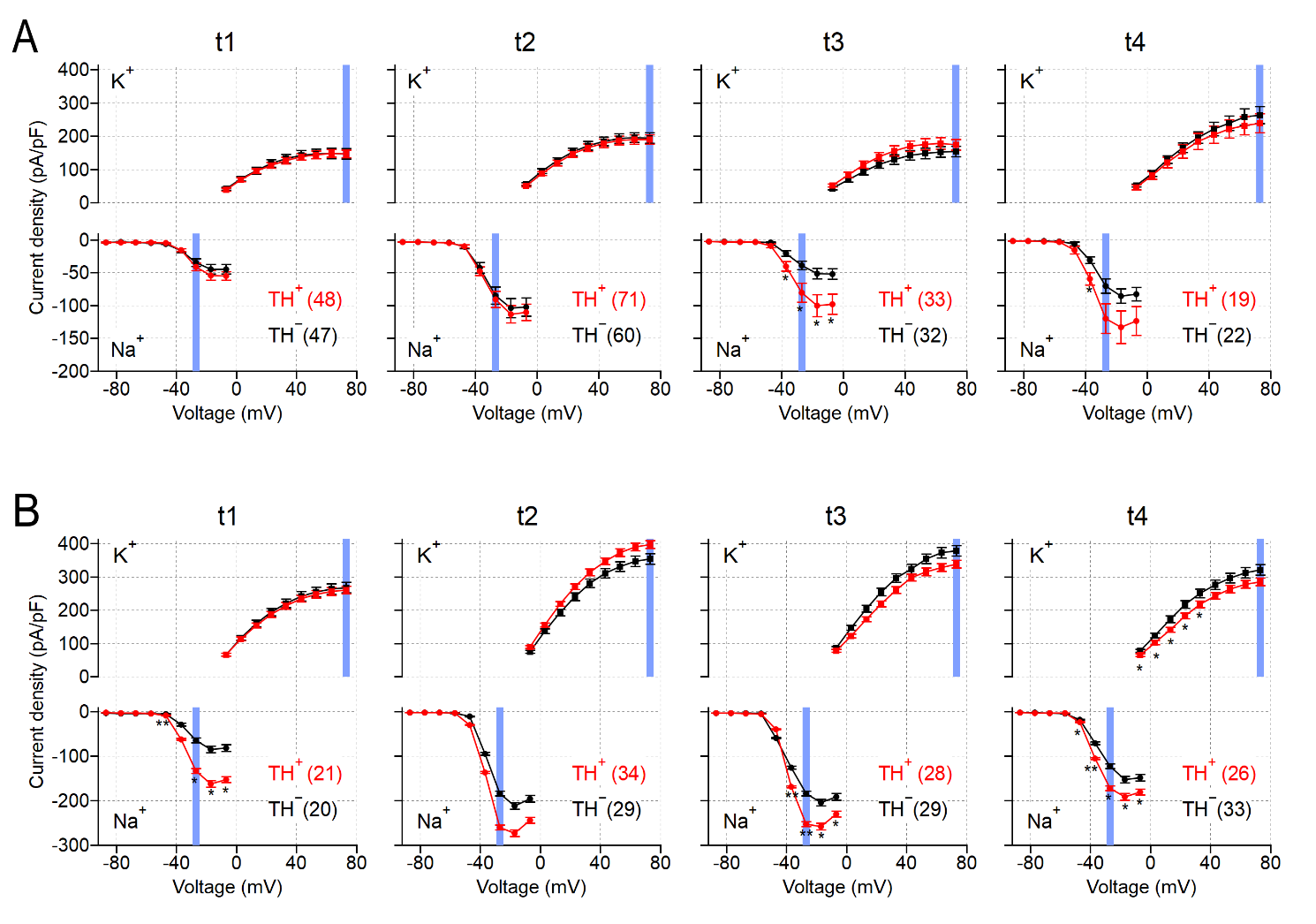
**

**Supplementary Figure 2.** Functional expression of Na_V_ and K_V_ channels in NPC- and iPSC-derived dopaminergic cultures. **(A)** Mean inward (*bottom*) and outward (*top*) current densities as a function of test pulse voltage, obtained from TH^−^ (black) and TH^+^ (red) NPC-derived neurons at specified time points (t1-4). Error bars indicate s.e.m., the numbers of cells analyzed in each timing group, *n*, is provided in parentheses. Straight lines connect means for clarity. Vertical blue lines at −27 mV and 73 mV mark data points used in Fig. 2C to analyze the development of Na_V_ and K_V_ channel-specific current components, respectively, during neuronal differentiation. **(B)** Identical set of experiments as in *A*, obtained from TH^−^ (black) and TH^+^ (red) iPSC-derived neurons. Statistical significance: ***P* < 0.01, **P* < 0.05 using a two-tailed Mann-Whitney *U*-test.


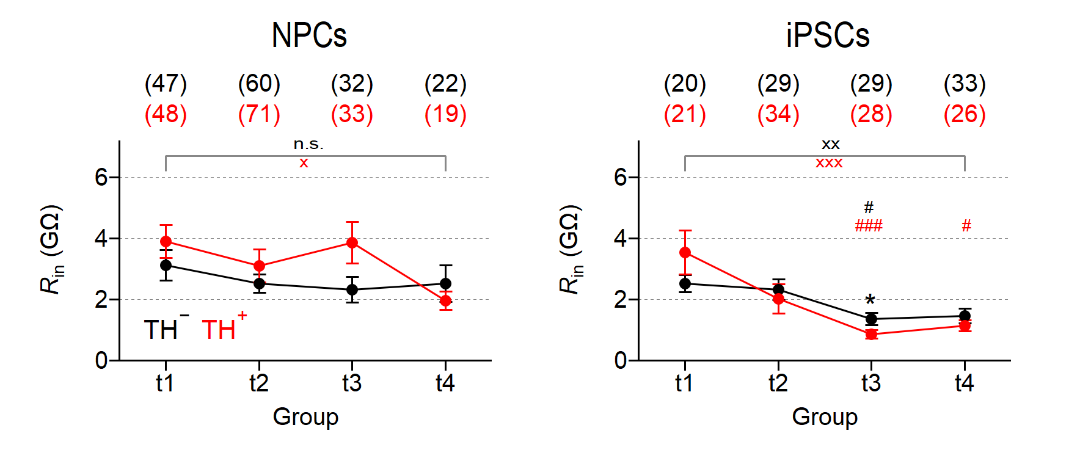


**Supplementary Figure 3.** Development of membrane input resistance of NPC- and iPSC-derived neurons. *R*_in_ was calculated for TH^−^ (black) and TH^+^ (red) NPC- (*left*) and iPSC-derived (*right*) neurons as the inverse of the linear slope of current responses to brief depolarizing voltage pulses to −87, −77, and −67 mV. Holding potential was −107 mV, leak currents were not subtracted. Data points are means ± s.e.m. with the number of experimental replicates, *n*, indicated in parentheses. Straight lines connect means for clarity. Significance between pairs of data was tested with a two-tailed Mann-Whitney *U*-test: **P* < 0.05 for testing TH^−^ against TH^+^ neurons from the same line; ^xxx^*P* < 0.01, ^xx^*P* < 0.01, ^x^*P* < 0.05 for testing data obtained at t1 against data obtained at t4; ^###^*P* < 0.001, ^#^*P* <­ 0.05 for testing data obtained from NPCs against data obtained from iPSCs; n.s., not significant.


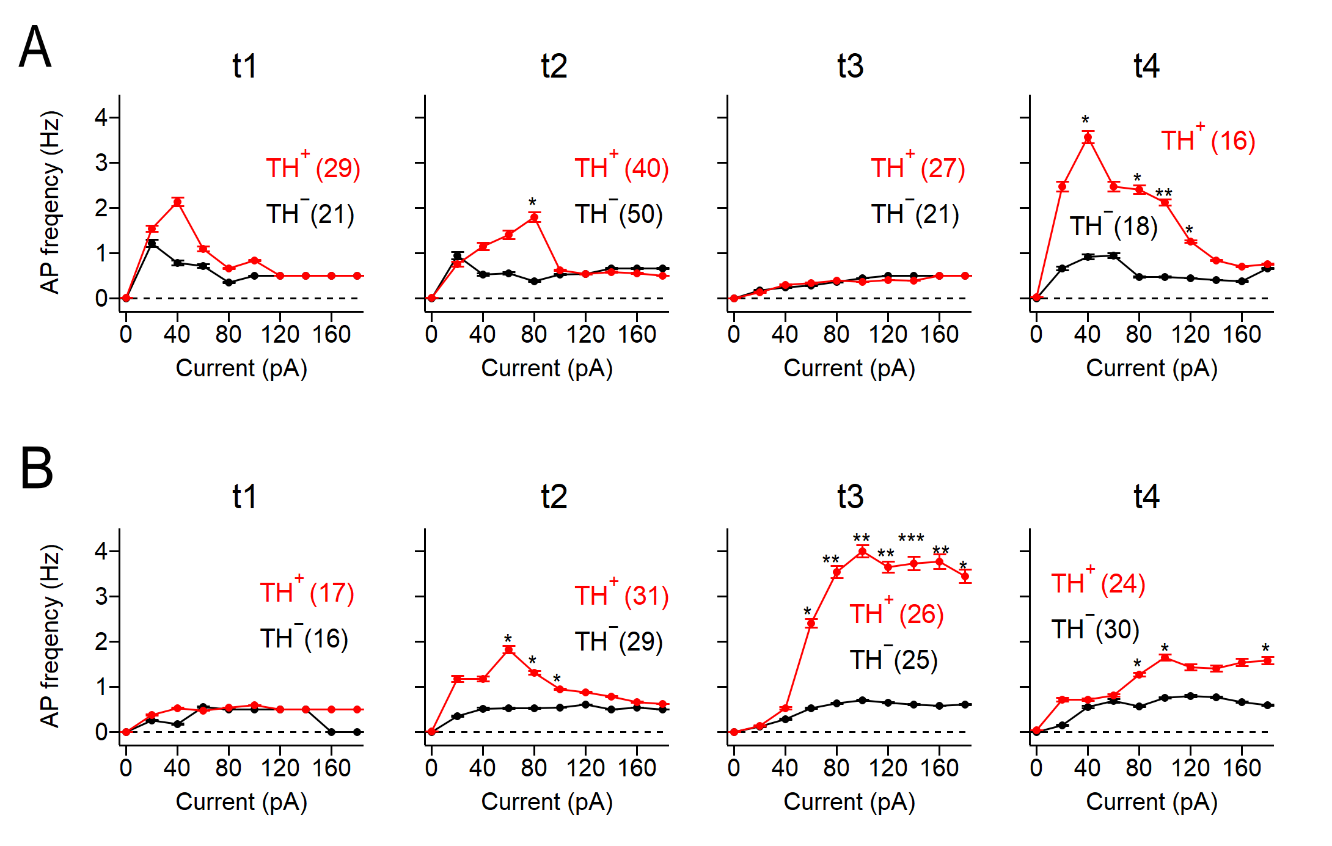


**Supplementary Figure 4.** Action potential firing frequencies of TH^−^ (black) and TH^+^ (red) neurons derived from NPCs **(A)** or iPSCs **(B)** in response to 2-s current injections ranging from 0 to 180 pA, at indicated time points (t1-t4). Data points are means ± s.e.m. with the number of experimental replicates, *n*, indicated in parentheses. Straight lines connect means for clarity. Asterisks indicate significant differences between TH^−^ (black) and TH^+^ (red) cells based on a two-tailed Mann-Whitney *U*-test: ^***^*P* < 0.01, ^**^*P* < 0.01, ^*^*P* < 0.05. Only cells that responded to the stimulation paradigm with at least one action potential were considered.


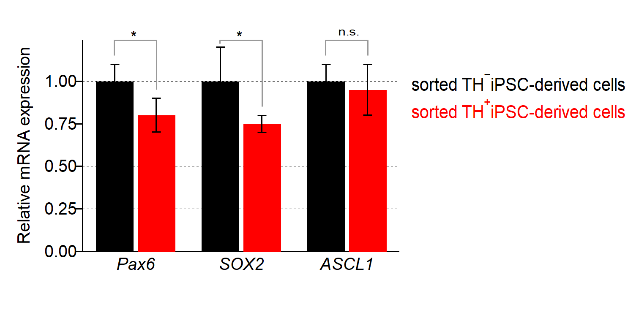


**Supplementary Figure 5.** Relative mRNA levels of neuronal progenitor markers Pax6, SOX2, and ASCL1 in TH^−^ (black) and TH^+^ (red) iPSC-derived neurons obtained after cell sorting by FACS at day 50 of differentiation. β−Actin mRNA served as a reference. Bars represent means ± s.e.m. of two independent experiments. Significance indicators refer to a Student's *t*-test: **P* < 0.05; n.s., not significant.

**Supplementary Table 1.** List of oligonucleotides used for quantitative PCR analysis.

|  | **Target** | **Forward / Reverse primer (5′-3′)** |
| --- | --- | --- |
| **Pluripotency markers (qRT-PCR)** | GDF3 | AAATGTTTGTGTTGCGGTCA / TCTGGCACAGGTGTCTTCAG |
|  | NANOG | TGAACCTCAGCTACAAACAG / TGGTGGTAGGAAGAGTAAAG |
|  | OCT4 (endogenous) | CCTCACTTCACTGCACTGTA / CAGGTTTTCTTTCCCTAGCT |
|  | SOX2 (endogenous) | CCCAGCAGACTTCACATGT / CCTCCCATTTCCCTCGTTTT |
| **NPC markers (qRT-PCR)** | Nestin | GCGTTGGAACAGAGGTTGGA / TGGGAGCAAAGATCCAAGAC |
|  | Pax6 | GTCCATCTTTGCTTGGGAAA / TAGCCAGGTTGCGAAGAACT |
|  | MSI1 | AGTCCTCCCCGAGCTTACAG / CATCGTCCAGGGGTGAGAG |
|  | ASCL1 | GGAGTTGGTATTCTCTCCCC / CTAAAGATGCAGGTTGTGCG |
| **Housekeeping gene (qRT-PCR)** | ACTB | TGAAGTGTGACGTGGACATC / GGAGGAGCAATGATCTTGAT |

# Supplementary References

Kriks S., Shim J.W., Piao J., Ganat Y.M., Wakeman D.R., Xie Z.*, et al.* (2011) Dopamine neurons derived from human ES cells efficiently engraft in animal models of Parkinson's disease. *Nature* **480**(7378): 547.

Reinhardt P., Glatza M., Hemmer K., Tsytsyura Y., Thiel C.S., Hoing S.*, et al.* (2013) Derivation and expansion using only small molecules of human neural progenitors for neurodegenerative disease modeling. *PLoS One* **8**(3): e59252.
